# Deep Brain Stimulation and Gene Expression Alterations in Parkinson’s Disease

**Published:** 2016-06-01

**Authors:** A. Mohammadi, A.R. Mehdizadeh

**Affiliations:** 1 Neuroscience Research Center, Baqiyatallah University of Medical Sciences, Tehran, Iran; 2 Department of Medical Physics, School of Medicine, Shiraz University of Medical Sciences, Shiraz, Iran

Parkinson disease (PD) is a neurobiological disorder caused by the death of dopaminergic neurons in the substantia nigra pars compacta (SNc). PD is typically characterized by features including rigidity, tremor, stiffness, and bradykinesia, as well as walking problems. Although, brain imaging and electroencephalography (EEG) is used in the primary evaluation of neurological disorders[1, 2], but it’s unfortunate that PD symptoms appear when approximately 60% of dopaminergic neurons have been destroyed[3]. The discovery of mutations in the genes for parkin (PARK2), DJ-1 (PARK7), PTEN-induced putative kinase 1 (PINK1), α-synuclein (SNCA), Ubiquitin Carboxyl-Terminal Esterase L1 (UCHL1), and Leucine-rich repeat kinase 2 (LRRK2) has made a unique glance into the mechanisms responsible for the etiology of PD[4]. Deep brain stimulation (DBS) is a neuroengineering procedure introduced in 1987 as a surgical handling for movement disorders specially the enervating symptoms of PD[5, 6]. Although, DBS has now been extensively considered as a lucrative method to patients whose medications have intensive signs cannot be sufficiently controlled with drugs, but it can improve movement disorders and the patient’s quality of life.

Traditionally, the subthalamic nucleus (STN), globus pallidus interna (GPi) and ventral intermediate nucleus of the thalamus (VIM) are three targets for DBS in PD. Whereas STN stimulation improves tremor, motor scores and some other dysfunctions, but there is also the potential for neurobiological and psychiatric side-effects, including gene expression alteration, hallucinations, depression, hyper-sexuality, apathy and cognitive dysfunction. Despite its noteworthy therapeutic potency, the exact mechanisms underlying the therapeutic effects of DBS haven’t been determined[7]. There are at least four hypotheses to explain the mechanisms of DBS including Synaptic depression[8], Synaptic inhibition[9], Depolarization blockade[10], and Stimulation-induced disruption of pathological network activity[11]. Regardless of the unclear mechanism of DBS, changes in crucial genes involved in Parkinson’s disease are very important. Although, it has been reported that high-frequency stimulation of the STN (STN HFS) increase striatal dopamine release and improve motor symptoms in intact rats[12, 13], but STN HFS can cause alteration of the genome of an organism which could be useful or harmful as well. For instance, STN HFS upregulate GAD67 mRNA expression in the substantia nigra pars reticulata and entopeduncular nucleus, c-fos in the STN, substance P and enkephalin in the striatum[14], Tyrosine Hydroxylase in the SNc and striatum, PMCH, IGF-2, IGFBP2, USAG1 and F5 in the basal ganglia[15], as well as downregulate of cytochrome oxidase subunit I (CoI) in the STN, calcium / calmodulin-dependent protein kinase type IIA (CaMKIIa), Homer 1, Ania1, KCNC3, Sv2b, TULIP1, LOC81816, CDH22 and IRSp53 in the basal ganglia[14, 15].

Visanji and colleagues (2015) reported that STN-DBS significantly transformed eight genes (Vps33b, Ppp1r3c, Mapk4, Sorcs2, Neto1, Abca1, Penk1, and Gapdh) in DRD2 striatopallidal medium spiny neurons (MSNs) and two overlapping genes in DRD1a MSNs (Penk1 and Ppp1r3c) concerned in the molecular mechanisms of STN-DBS[16]. Soreq et al (2013) demonstrated that DBS modulates nonsense-mediated RNA decay in Parkinson’s patient’s leukocytes[17].

Also, STN HFS induces a significant rise of extracellular GABA levels in the SN and glutamate in the GP and SN[18]. Spieles-Engemann and coworkers (2011) reported that STN-DBS increases brain derived neurotrophic factor (BDNF) in the nigrostriatal system and primary motor cortex which may be linked to glutamate transmission[19]. Altogether, several open questions remain as to the detailed mechanisms and functions of DBS; particularly in terms of the neurochemical and genomic effects of DBS in the brain. However, many of the cellular and molecular studies are needed to determine the exact mechanism of DBS. 
